# Mycolactone as Analgesic: Subcutaneous Bioavailability Parameters

**DOI:** 10.3389/fphar.2019.00378

**Published:** 2019-04-12

**Authors:** Jérémie Babonneau, Dimitri Bréard, Marie-Line Reynaert, Estelle Marion, David Guilet, Jean-Paul Saint André, Anne Croué, Priscille Brodin, Pascal Richomme, Laurent Marsollier

**Affiliations:** ^1^Equipe ATIP AVENIR, CRCINA, INSERM, University of Nantes, University of Angers, Angers, France; ^2^EA921 SONAS, SFR4207 QUASAV, University of Angers, Université Bretagne-Loire, Angers, France; ^3^CNRS, Inserm, CHU Lille, Institut Pasteur de Lille, U1019 – UMR8204 – CIIL – Center for Infection and Immunity of Lille, University of Lille, Lille, France; ^4^Laboratoire d’Anatomie Pathologique, Angers, France

**Keywords:** *Mycobacterium ulcerans*, mycolactone, analgesia, bioavailability, biological action

## Abstract

*Mycobacterium ulcerans* is the bacillus responsible for Buruli ulcer, an infectious disease and the third most important mycobacterial disease worldwide, after tuberculosis and leprosy. *M. ulcerans* infection is a type of panniculitis beginning mostly with a nodule or an oedema, which can progress to large ulcerative lesions. The lesions are caused by mycolactone, the polyketide toxin of *M. ulcerans*. Mycolactone plays a central role for host colonization as it has immunomodulatory and analgesic effects. On one hand, mycolactone induces analgesia by targeting type-2 angiotensin II receptors (AT_2_R), causing cellular hyperpolarization and neuron desensitization. Indeed, a single subcutaneous injection of mycolactone into the mouse footpad induces a long-lasting hypoesthesia up to 48 h. It was suggested that the long-lasting hypoesthesia may result from the persistence of a significant amount of mycolactone locally following its injection, which could be probably due to its slow elimination from tissues. To verify this hypothesis, we investigated the correlation between hypoesthesia and mycolactone bioavailability directly at the tissue level. Various quantities of mycolactone were then injected in mouse tissue and hypoesthesia was recorded with nociception assays over a period of 48 h. The hypoesthesia was maximal 6 h after the injection of 4 μg mycolactone. The basal state was reached 48 h after injection, which demonstrated the absence of nerve damage. Surprisingly, mycolactone levels decreased strongly during the first hours with a reduction of 70 and 90% after 4 and 10 h, respectively. Also, mycolactone did not diffuse in neighboring skin tissue and only poorly into the bloodstream upon direct injection. Nevertheless, the remaining amount was sufficient to induce hypoesthesia during 24 h. Our results thus demonstrate that intact mycolactone is rapidly eliminated and that very small amounts of mycolactone are sufficient to induce hypoesthesia. Taken together, our study points out that mycolactone ought to be considered as a promising analgesic.

## Introduction

*Mycobacterium ulcerans* is the bacillus responsible for Buruli ulcer, an infectious disease and the third most important mycobacterial disease worldwide, after tuberculosis and leprosy. *M. ulcerans* infection is a type of panniculitis beginning mostly with a nodule or an oedema, which can progress to large ulcerative lesions (Vincent et al., 2014). The lesions are caused by mycolactone, the polyketide toxin produced by *M. ulcerans* (George et al., 1999). Mycolactone exerts a wide range of effects and disrupts fundamental cellular processes, like cell adhesion and signaling pathways (Hall and Simmonds, 2014; see also Sarfo et al., 2016 for review). Mycolactone pathogenicity was shown to be mediated by the blockade of proteins translocation into the endoplasmic reticulum (Hall et al., 2014). In particular, Sec61 translocon was identified as a major target of mycolactone and its inhibition has critical consequences on the immune system (Baron et al., 2016). Importantly, unlike the lesions of many other diseases, Buruli ulcer lesions cause little or no pain (Johnson et al., 2005; Sizaire et al., 2006). It was first suggested that this painlessness results directly from nerve destruction at late stages of the disease (Goto et al., 2006; En et al., 2008). However, this may not explain the analgesic effects observed at earlier stages, before the occurrence of tissue destruction. It has also been proposed that Sec-61 blockade by mycolactone could contribute to analgesia by suppressing inflammation (Isaac et al., 2017). Interestingly, we previously reported that mycolactone displayed hypoesthesic properties in a non-inflammatory context (Marion et al., 2014; Song et al., 2017). Indeed, we show that naïve mice that were inoculated with mycolactone were less sensitive to a pain stimulus using the Hargreaves plantar pain test and that this effect was dependent on the type-2 angiotensin II receptors (AT_2_R). Correlating with this, mycolactone induced a sustained hyperpolarization in sensory neurons involving the release of potassium through TRAAK channels in an AT_2_R dependent manner. Moreover, a single subcutaneous injection of mycolactone into the mouse footpad induced a long-lasting hypoesthesia up to 48 h (Marion et al., 2014). The return to normal sensitivity after this period also proved clearly the absence of nerve destruction in this mouse model. It was hypothesized that this long-lasting hypoesthesia may result from the persistence of large amounts of mycolactone locally after the injection, which could be probably due to its slow elimination from tissues (Sarfo et al., 2011). To test this hypothesis, we investigated here the correlation between the amount of mycolactone in tissues and its biological effect by measuring hypoesthesia. Nociception assays in mice inoculated with various doses of mycolactone that did not cause any tissular damages nor inflammatory responses, were performed using the Hargreaves plantar test method. In parallel, the amounts of mycolactone released in cutaneous tissues were determined by ultra performance liquid chromatography-mass spectrometry (UPLC-MS). We report here that the maximum analgesic effect of mycolactone was observed at a time post-injection, when mycolactone amounts remaining in the tissue were very low nonetheless similar to the ones obtained in oedematous tissues of mice infected with *M*. *ulcerans*.

## Materials and Methods

### Animal Experimentation

All animal experiments were performed in accordance with national (articles R214-87 to R214-90 from the French “rural code”) and European guidelines (directive 2010/63/EU of the European Parliament and of the Council of September 22, 2010 on the protection of animals used for scientific purposes). All protocols were approved by the Ethics Committee of the Pays de la Loire region under protocol CEEA 2015121410496026. Animals were maintained under specific pathogen-free conditions in the animal house of Angers University Hospital, Angers, France (agreement A 49 007 002).

### Mycolactone Extraction and Purification

Mycolactone A/B was purified from *M. ulcerans* (strain 1615) extracts as previously described (George et al., 1999; Marion et al., 2012). Mycolactone purity and concentration were determined by ultra performance liquid chromatography (UPLC), as previously described (Marion et al., 2012). The concentration of the mycolactone solution was adjusted to 3 mg/mL in absolute ethanol and the solution was stored in the dark in glass amber vials.

### Mouse Models

#### Mycolactone Injection

For nociception assays, various amounts of mycolactone that do not induce tissular damages or inflammatory response were injected (1, 2, and 4 μg) subcutaneously into the right footpad of seven-week-old female Balb/c mice (Charles River Laboratories, Saint-Germain-Nuelles, France). Mycolactone was diluted in 15 μL corn oil with 8% ethanol and the vehicle was 15 μL corn-oil with 8% ethanol.

For the determination of mycolactone levels in tissues and blood, 4 μg of mycolactone diluted in 15 μL corn oil were injected subcutaneously into the right footpad of the mouse. Mice were killed at *t* = 0, 4, 6, 24, and 48 h, and footpads were dissected for lipid extraction. The quantity of mycolactone in the tissues of *M. ulcerans*-infected mice at the oedema stage was determined by lipid extraction from mouse tail tissues.

#### *M. ulcerans* Inoculation

Bacterial suspensions were prepared as previously described (Marsollier et al., 2003). The cell density was adjusted to 5 × 10^5^ acid-fast bacilli/ml for inoculation (20 μL) into the tail of six-week-old consanguineous BALB/c females (Charles River Laboratories, Saint-Germain-Nuelles, France).

Bacterial load was evaluated by CFU (Colony Forming Units). Briefly, tails from five *M. ulcerans* infected mice at the oedema stage of infection were excised and individually homogenized in sterile water. Following decontamination (Marsollier et al., 2003), tissues samples were inoculated onto Löwenstein–Jensen slants (BD Biosciences), and CFU were counted after 15 weeks of incubation, allowing assessment of very low bacterial load.

#### Pain Receptivity Assay

Pain receptivity was assessed by Hargreaves method (Hargreaves et al., 1988), with a plantar test instrument (Ugo Basile, Gemonio, Italy). All animals were acclimatized to the apparatus for 15 min on at least three consecutive days before the experiment and for 10 min before each test. The response to an infrared stimulus focused on the right footpad was measured. The intensity of the stimulus was set at 35% and a cut-off time of 30 s was applied to prevent the lesioning of mouse tissues by the stimulus. The time from the start of the stimulus to paw retraction (latency) was recorded automatically. The flicking/licking of the paw was confirmed by visual observation. Results were expressed as the ratio of the latencies of the response to a heat stimulus between mycolactone-treated groups and vehicle-treated groups, at 2, 4, 6, 24, and 48 h after injection.

### Lipid Extraction From Mouse Tissues

Once the mouse had been killed, its right footpad (for mycolactone amounts assessment at different time after injection and for assessment of mycolactone diffusion) or tail (for inoculation with *M. ulcerans*) was removed, cut into small pieces and placed in a 15 mL centrifuge tube (Greiner Bio-one, Les Ulis, France). For both experiments, 2 mL methanol was added and the tissues were ground with a Potter-Elvehjem pestle (Dutscher Scientific, Issy-les-Moulineaux, France; 045080). The suspension was transferred to a 15 mL solvent-resistant centrifuge tube (VWR, Fontenay-sous-bois; 525-0401) and 4 mL chloroform was added. The mixture was then incubated overnight, in the dark, with shaking. Folch’s extraction was completed by adding 600 μL distilled water and shaking the mixture vigorously. The mixture was then centrifuged at 4000 × *g* for 10 min, to separate the aqueous and organic phases. The organic phase, containing the lipids, was transferred to a glass tube and allowed to dry in a centrifugal evaporator. The remaining material was recovered in 200 μL ice-cold acetone for phospholipid precipitation. The suspension was centrifuged for 10 min at 4000 × *g.* The supernatant was collected and stored at -20°C, in the dark, in amber glass vials, until use.

### Lipid Extraction From Total Blood

Six hours after the subcutaneous injection of 4 μg mycolactone into the right footpad, 1.5 mL total blood were collected in a 15 mL solvent-resistant centrifuge tube. Lipids were extracted by Folch’s method, as described above.

### Histological Study

Mouse tissues (footpads) were fixed by incubation in 10% neutral buffered formalin for 15 days for routine embedding in paraffin. Sections (5 μm thick) were cut and stained with Haematoxylin/Phloxin/Saffran.

### Mycolactone Determination by Liquid Chromatography Coupled to Tandem Mass Spectrometry

#### UPLC-MS Instrumentation and Conditions

Similarly as was described by others (George et al., 1999; Hong et al., 2003; Sarfo et al., 2011), liquid chromatography (LC) was performed with a Thermo Accela High-Speed LC system equipped with a refrigerated autosampler and a column oven (Thermo Fisher Scientific, Gometz-le-Châtel, France). The LC system was coupled to a photodiode array detector and a mass spectrometry detector in series. Samples were injected, with a 5-μL loop in partial loop mode (1 μL injected), onto a Hypersil GOLD C8 column (100 × 2.1 mm; 1.9 μm; Thermo Fisher Scientific). The column was heated at 30°C. A gradient of water (A) and acetonitrile (ACN; B) was used (60–100% B in 10 min, then 100% B for 5 min), at a flow rate of 300 μL/min. Mass spectrometry (MS) experiments were performed with a Thermo TSQ Quantum Access MAX machine equipped with a heated electrospray interface (HESI) operating in positive ionization mode (Thermo Fisher Scientific). The mycolactone A/B standard was infused into the electrospray ion source at a concentration of 0.5 mg/mL in ethanol, with a syringe pump operating at a flow rate of 5 μL/min, together with 250 μL/min water/ACN mixture (40/60), to optimize the ESI conditions (spray voltage, 4500 V; vaporizer temperature, 350°C; sheath gas pressure, 40 arbitrary units (au); ion sweep gas pressure, 0 AU; auxiliary gas pressure, 15 AU; capillary temperature, 375°C; tube lens offset, 35 V; skimmer offset, 0 AU) and the selected reaction monitoring (SRM) conditions (collision gas pressure, 1.8 mTorr; collision energy, 42 V). The nebulisation gas used was nitrogen (N_2_) and the collision gas was argon (Ar). Data were acquired and processed with Xcalibur software 2.1 (Thermo Fisher Scientific).

#### Preparation of Standard Solutions and Matrix Effect

A primary stock solution was prepared by dissolving 0.8 mg mycolactone A/B standard in 1 mL ethanol. The solution was stored at -20°C. The primary stock solution was diluted in ethanol to prepare the calibration solutions (5000, 2500, 500, 100, 50, 25, 10.0, and 5.0 ng/mL). The peak areas obtained for blank samples spiked with mycolactone A/B at the end of sample preparation were compared with those recorded for the corresponding calibration solutions.

#### Validation of the Method

The method was developed with mycolactone A/B prepared as described above. The linearity, limit of detection (LOD), limit of quantification (LOQ) and repeatability of the method were evaluated. The linearity of the calibration curve was assessed by injecting serial dilutions of calibration solutions in six replicates. Residuals (difference between nominal concentration and calculated concentration according to the linear model) and their distribution (normally distributed around the mean) were checked. Similarly, LOD and LOQ were estimated with a final criterion signal-to-noise ratio (S/N) of 3 and 10, respectively. The coefficient of the peak areas was taken as a measurement of precision. The repeatability of the method, expressed as the relative standard deviation (RSD in %), was estimated by measuring mycolactone A/B levels in several replicates (*n* = 4 LC injections) of a lipid extract (*n* = 4 sample replicates).

### Statistical Analysis

GraphPad Prism 7 was used to compare the test group means with the vehicle group means at each time point, by two-way ANOVA followed by a Dunnett’s multiple comparison test.

## Results

### Mycolactone Induces Long-Lasting Dose-Dependent Hypoesthesia

**FIGURE 1:**
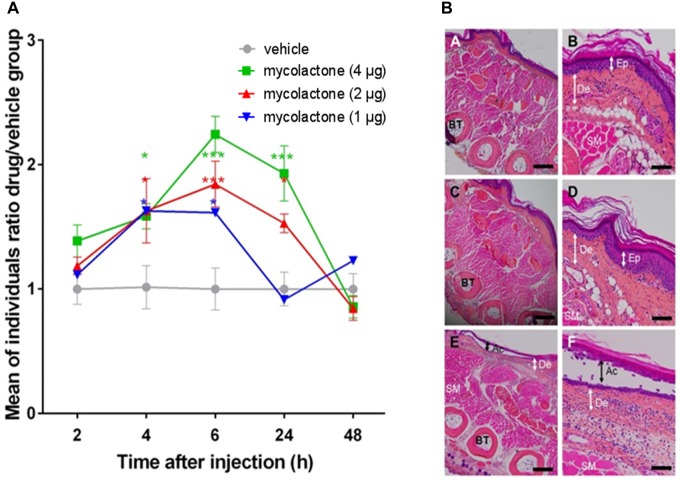
Mycolactone induces hypoesthesia in absence of inflammation. **(A)** The hypoesthesia caused by mycolactone was monitored by measuring the nociceptive reflex in mice after injecting various amounts of mycolactone (1, 2, and 4 μg) using the Hargreaves plantar test. The hypoesthesia expressed as the ratio of the latencies of the response to a heat stimulus between each mycolactone-treated group and the control group (treated with vehicle), at various times after injection. Mean ± SEM for 7 mice per group tested. **(B)** Histological sections of mouse foot pad stained with Haematoxylin/Phloxin/Saffran 48 h after inoculation with **(A,B)** vehicle alone, **(C,D)** 4 μg of mycolactone and **(E,F)** 8 μg of mycolactone. No inflammatory response was observed in tissue inoculated with vehicle or with 4 μg of mycolactone. While, at 8 μg injected mycolactone, an important inflammatory response and acantholysis were observed **(E,F)**. De: Dermis, Ep: Epidermis, BT: Bone Tissue, SM: Striated Muscle, Ac: Acantholysis. Scale bars, 500 μm **(A,C,E)**, 100 μm **(B,D)**, 150 μm **(F)**.

Various doses of mycolactone were injected subcutaneously into mouse footpads and hypoesthesia was assessed with Hargreaves plantar test 2, 4, 6, 24, and 48 h after injection (Figure 1A). Hypoesthesia was measured for doses of 1 to 4 μg mycolactone per footpad. Tissue examination by histological approaches at 2, 24, and 48 h showed that the concentrations of mycolactone inoculated did not induce any tissue damage nor a cellular inflammatory response (Figure 1B). Putative toxicity after longer time periods was also assessed (72 h, 7 and 15 days) and no side effects of mycolactone were observed (data not shown). Nevertheless, we cannot completely rule out that there might be a risk for potential side effects that we did not noticed. For comparison, a higher dose of 8 μg did induce tissue damage, hence highlighting the importance of a proper control of the dose used in *in vivo* experiments. Two hours after injection, the latency time upon a heat stimulus increased by 11% for the dose of 1 μg dose, 19% for 2 μg and 39% for 4 μg, relative to vehicle. Four hours after injection, latency had continued to increase in all the mycolactone-treated groups, by a maximum of 60% (*p* < 0.001) relative to vehicle. Between 4 and 6 h, hypoesthesia (latency) remained stable but significantly different from the control (*p* < 0.05) in the 1 μg mycolactone group, whereas the hypoesthesia induced by mycolactone increased for the groups receiving 2 or 4 μg (Figure 1A). Maximal hypoesthesia was reached at 6 h with an 80% increase for 2 μg and 120% increase for 4 μg relative to vehicle, respectively. After 6 h, hypoesthesia gradually decreased, for all doses of mycolactone injected. At 24 h, the group receiving 1 μg of mycolactone displayed no hypoesthesia, whereas it remained significant in the groups receiving 2 and 4 μg, with increases in latency of 53% (*p* < 0.05) and 93% (*p* < 0.001^∗^), respectively, relative to vehicle (Figure 1A). After 48 h, no hypoesthesia was detected in comparisons of the control group with the groups receiving the vehicle (Figure 1A). These findings demonstrate that the hypoesthesia of mycolactone is dose-dependent, and that it develops slowly, peaking at 6 h after injection, and lasts 24–48 h for doses of 2 and 4 μg. The return to the basal state 48 h after injection demonstrates the absence of nerve damage at this time point (Figure 1A). Based on our results, we suggest that, 6 h after mycolactone injection, the residual mycolactone remaining in the tissues is located close to the site of injection and that the long latency (4 h) required to obtain significant hypoesthesia with may be due to the time required for the toxin to diffuse and reach its cellular target (AT_2_R).

### Development of the Mass Spectrometry Approach for Quantifying Mycolactone in Tissues: Limit of Detection (LOD) and Efficiency of the Extraction Method

Sensitive methods for mycolactone quantification are required, to evaluate the relationship between mycolactone dose and the intensity of the induced hypoesthesia. We evaluated a detection method based on liquid chromatography coupled to mass spectrometry for the detection and quantification of mycolactone in the complex liquid matrix extracted from mouse tissues described in a previous study (Sarfo et al., 2013).

We first used a purified mycolactone solution to determine the limits of detection/quantification by liquid chromatography-tandem mass spectrometry:

#### UPLC-MS Optimization for Mycolactone Characterization and Quantification

The optimization of chromatographic conditions gave retention times of 7.6 and 8.4 min for mycolactones A and B, respectively, with a total run time of 15 min (George et al., 1999; Marion et al., 2012). Mycolactone A/B is an equilibrated mixture of geometric Z-Δ^4′-5′^ (mycolactone A, major compound) and E-Δ^4′-5′^ (mycolactone B, minor compound) isomers. Mycolactones A and B generated an overlapping two-peak cluster and were, therefore, quantified together. MS analyses of the mycolactone A/B standard revealed the presence of an ion with a mass-to-charge ratio (m/z) of 765.5, corresponding to sodium adduct [M+Na]^+^, as the main component. Selected reaction monitoring (SRM) of this ion yielded an ion with a m/z of 429.2, corresponding to the core lactone ring of mycolactones (Hong et al., 2003). This enabled accurate identification with a very good signal-to-noise ratios. Indeed, mycolactone A/B was readily quantified even when barely detectable in the total ion current (TIC).

#### Method Validation

The method described here allows the detection and quantification of mycolactone simply, rapidly and specifically in a matrix of mouse tissue lipids. Unambiguous resolution was achieved for mycolactone with a gradient of water and acetonitrile (60–100% B in 10 min, then 100% B for 5 min) as the mobile phase. Separation was achieved on a reverse-phase Hypersil GOLD C8 column heated at 30°C, with a flow rate of 300 μL/min. Under these conditions, the mycolactone A/B analytes were fully separated in 9 min, with a mean retention time of 7.6 min for mycolactone A and 8.4 min for mycolactone B, forming the characteristic two-peak curve of mycolactone A/B (Supplementary Figure S1) and the references (Marion et al., 2012).

This method was validated for the quantification of mycolactone A/B, in accordance with performance criteria, by assessing precision, linearity, limit of detection (LOD) and limit of quantification (LOQ). We used best-fit linear regression to determine the concentration-detector relationship. We obtained a mean correlation coefficient of 0.9989 with SRM detection from 5 pg to 5 ng, in accordance with widely used guidelines [Guidelines for the Validation of Chemical Methods for the FDA FVM Program, Food and Drug Administration (2015)].

The precision of the method was evaluated, including sampling (*n* = 4) and chromatographic measurement (*n* = 4), the associated RSD value was 12.3%.

The LOD and LOQ were 1 and 5 pg, respectively (Supplementary Figure S1).

We then assessed the efficiency of mycolactone extraction from tissue, by injecting 4 μg of mycolactone, which is a dose inducing the strongest and longest lasting effect (Figure 1) into mouse footpads (*n* = 5). The mice were immediately killed and their footpads were rapidly dissected and used for total lipid extraction. The mycolactone content of the lipid extract was quantified by liquid chromatography coupled to mass spectrometry. The mean percentage of mycolactone amount extracted from spiked footpads was 10.5% (6.2 to 18.8%), corresponding to the recovery of 421.47 ng (±95.4 ng SEM) of the 4 μg mycolactone initially injected per footpad (Table 1). Extraction efficiency (10.5%) therefore appears very low, but the value obtained was similar to those reported in other studies (Sarfo et al., 2013; Converse et al., 2014). It is therefore necessary to take this low extraction capacity into account when evaluating the amount of mycolactone present in the tissues.

**Table 1:** Mycolactone recovery after extraction from tissues.

	Tissue sample 1	Tissue sample 2	Tissue sample 3	Tissue sample 4	Tissue sample 5	*Mean*	*SD*	SEM
Mycolactone (ng/g)	248.6	319.4	751.9	269.0	518.4	421.5	213.4	95.41
Mycolactone recovery (%)	6.22	7.99	18.80	6.72	12.96	10.5	11.6	2.38


*We injected 4 μg of mycolactone subcutaneously into the footpad and then quantified mycolactone in the total lipid fraction extracted from the footpad*.

### The Amount of Mycolactone in Tissues Decreases Rapidly After Subcutaneous Injection

**FIGURE 2:**
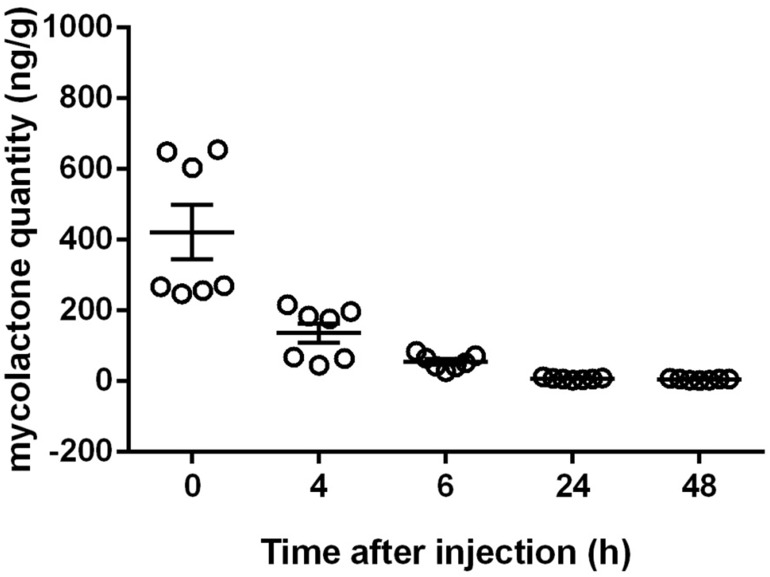
Quantification of mycolactone after tissue injection. Amounts of mycolactone recovered from footpad total lipid extract by LC-MS/MS (ng/g) are represented as scatterplots at *t* = 0, 4, 6, 24, and 48 h after the injection of 4 μg mycolactone (mean ± SEM of 7 mice is also represented in the figure). At *t* = 0 h, 4 μg of mycolactone was injected into the footpad.

As mycolactone appeared to induce analgesia in a dose-dependent manner, we monitored the changes in mycolactone levels in mouse footpad over 48 h, hypothesizing that mycolactone levels in tissues would decrease slowly. We injected 4 μg of mycolactone subcutaneously into mouse footpad (*n* = 7). At *t* = 0, 4, 6, 24, and 48 h after injection, we extracted total lipids from the tissue into which the mycolactone was injected and then determined mycolactone concentration by UPLC-MS (Figure 2 and Table 2). The mean amount of mycolactone at *t* = 0 h was 421.7 ng/g (±61 ng), giving a recovery rate of 10.5% similar to the percentage reported above for the calibration experiments (Table 1). Mycolactone levels decreased strongly during the first few hours with only 136.3 ± 20 ng/g of the extractable injected mycolactone remaining after 4 h and 55 ± 6 ng/g remaining after 6 h (Table 2). After 24 h, 98.3% of the extractable injected dose was eliminated from the footpad, with only 7.4 ± 1 ng/g detected. At *t* = 48 h, 98.8% (5.3 ± 1 ng) of the initially extractable mycolactone had been eliminated (Figure 2 and Table 2). Taken together, this demonstrates that very small amounts of mycolactone were sufficient to induce hypoesthesia. The rapid initial decrease in mycolactone levels could be accounted for (i) the degradation of mycolactone locally by enzymatic or chemical reactions, (ii) the diffusion of mycolactone into the bloodstream and/or (iii) the diffusion of mycolactone into nearby tissues. These two latter hypothesis were further tested.

**Table 2:** Quantification of mycolactone in footpads.

Time after injection (h)	Amounts of mycolactone detected (ng/g) at the inoculation site Mean ± SEM	*SD*	Mycolactone recovery (%(mean))
0	421.7 ± 61	201.8	10.8
4	136.3 ± 20	73.1	
6	55 ± 6	20.1	
24	7.4 ± 1	3.1	
48	5.3 ± 1.9	1.9	


*Mycolactone was quantified in the total lipid fraction extracted from the footpad into which 4 μg mycolactone was injected*.

### Mycolactone Could Not Be Detected in Neighboring Skin Tissues

We inoculated mice footpad with mycolactone and then investigated the distribution of this molecule nearby the site of inoculation. Mycolactone was detected only in the inoculation site (Table 2). The direct diffusion of mycolactone in tissues therefore seems unlikely.

### Mycolactone Diffuses Into the Bloodstream Upon Direct Injection

We next investigated whether the decrease in mycolactone levels resulted from diffusion in the bloodstream away from the tissue into which it was injected.

We assessed the diffusion of mycolactone diffusion from tissue to blood, by inoculating six mice subcutaneously (footpad) with 4 μg mycolactone. Mycolactone was detected at 6 h in only three of six mice with a mean amount of 33.2 ng per millilitre (Table 3), clearly showing that mycolactone was able to diffuse into the bloodstream in small amounts. Thus, this suggests that some of the subcutaneously injected mycolactone diffused continuously from tissues to blood.

**Table 3:** Quantification of mycolactone (i) in bloodstream upon mycolactone inoculation in footpad and (ii) in tails after infection with *M. ulcerans*.

Mouse number	Mycolactone amounts in blood 6 h after injection in footpad (ng/mL)	Mycolactone amounts in tails infected with *M. ulcerans* at oedematous stage (ng/g)
1	19.2	9.6
2	32.8	16.4
3	47.6	23.8
4	<LOD	ND
5	<LOD	ND
6	<LOD	ND


*Mycolactone was quantified in the total lipid fraction in bloodstream 6 h after mycolactone injection (*n* = 6) and in the total lipid fraction extracted from the tail after infection with *M. ulcerans* at oedematous stage (*n* = 3). LOD: Limit of detection; ND: Not determined*.

### Mycolactone Does Not Diffuse Into the Bloodstream in the Context of an *M. ulcerans* Infection

We next investigated mycolactone levels at a stage of infection at which there is an analgesic effect but no tissue destruction, namely the oedematous stage (En et al., 2008; Marion et al., 2014). At this stage of infection, bacterial load (CFU) was estimated at 3.15 × 10^5^ and does not vary in the course of the 48 h experiment as the generation time is 3.5 days (Marsollier et al., 2003). We then determined the amount of mycolactone in *M. ulcerans*-infected tissues (tails) by extracting total lipids from three oedematous tissues. We showed that mycolactone levels in these tissues (*n* = 3) were low with a mean amount of 16.6 ng per gram (Table 3), which is consistent with previous studies (Converse et al., 2014; Sarfo et al., 2014; Marion et al., 2016a,b). We next evaluated the amounts of mycolactone present in the total blood of the same mice experimentally infected with *M. ulcerans* at the oedematous stage. To this end, 1.5 ml of blood from each of six mice was analyzed. We were unable to detect even trace amounts of mycolactone in the total blood of infected mice. This failure to detect mycolactone in the bloodstream may reflect the use of an inoculum of *M. ulcerans* that was too small. In conclusion, there is no proof yet that mycolactone diffuses in the blood in the context of *M. ulcerans* infection.

## Discussion

Pain represents currently the most common symptom for which medical attention is sought by patients (Melnikova, 2010). Available treatments have limited effectiveness and significant side-effects (Borsook et al., 2014; Stern and Roberts, 2016). In addition, analgesia duration is often low. Handling of pain remains though a major societal and humanitarian challenge.

Mycolactone is a toxin produced by *M. ulcerans* and responsible for the ulcerative skin lesions observed in patients suffering from Buruli ulcer (George et al., 1999; Vincent et al., 2014). Interestingly, mycolactone is also responsible for the painless character of the lesions it causes, at least at early stages of the disease (Sizaire et al., 2006). In mice models, mycolactone induces hypoesthesia with an extremely long-lasting effect (En et al., 2008; Marion et al., 2014).

Here, we investigated the correlation between hypoesthesia and amounts of toxin in local cutaneous tissues as well as in the blood stream. To address this issue, we first assessed the dose dependence of mycolactone on pain behavior. We injected mycolactone, at doses that had no impact on inflammation or on tissues into mouse footpads, for instance 1 to 4 μg per footpad. A correlation was found between dose and hypoesthesic effect, with the strongest and longest lasting effect recorded for the 4 μg dose, peaking 6 h after injection and remaining statistically significant until 24 h.

Unexpectedly, the maximum effect observed in nociception assays did not correlate with the maximum amount of mycolactone detected in mice footpads. For instance, only 30% of the extractable injected mycolactone remained after 4 h, to the time point at which we began to observe significant hypoesthesia. Six hours after mycolactone injection, the effect of hypoesthesia was maximal although mycolactone remaining amounts were around 10%. This time lag may be due to the diffusion time required for mycolactone to reach its cellular targets. It is also possible that mycolactone displays a high affinity for AT_2_R *in vivo*, which may account for this apparent paradox. Indeed, in support of this, we previously showed that blockade of AT_2_R signaling fully inhibited mycolactone-induced hypoesthesia in *in vivo* experiments relying on the use of the thermal pain Hargreaves test (Marion et al., 2014). Rather, Guenin-Macé and colleagues suggested that the inhibition of Sec61 activity by mycolactone could account for the hypoesthesic effect of mycolactone. The interaction between mycolactone and Sec61 was shown to promote cell death via a reticulum endoplasmic stress response triggering cell apoptosis (Ogbechi et al., 2018) but also to lead to an impaired production of key mediators of immune responses (Baron et al., 2016; Isaac et al., 2017). The mycolactone-mediated effect on the initiation of the stress response is rapid, within 1 h, which is similar to the hypoesthesic effect of mycolactone that is detected 2 h after mycolactone injection arguing for the involvement of such broad spanning pathway in the hypoesthesia (Ogbechi et al., 2018). However, it is important to emphasize that mycolactone-mediated Sec61 blockade was observed in an inflammatory context, after prolonged exposure of cells with the toxin (Isaac et al., 2017; Morel et al., 2018) or when cellular stress response pathways are studied. More experiments with an *in vivo* model of analgesia are thus needed. Another explanation would be that mycolactone is protected from degradation/elimination when bound to AT_2_R, and that this binding is established and active for long periods. Accordingly, slow elimination of mycolactone from tissues has been shown in Buruli ulcer patients (Sarfo et al., 2011). We can also assume that the degradation products of mycolactone can induce hypoesthesia, as reported by others that painkillers metabolites display potent analgesic properties (Cregg et al., 2013). The rapid decrease in mycolactone levels after the injection of this toxin into tissues was rather unexpected. Indeed, 6 h after inoculation, more than 90% of the mycolactone had disappeared. Interestingly, the remaining amount lies in the range of values obtained by mass spectrometry and reported for mycolactone in extracts from 4 mm biopsy specimens of nodules, oedemas and plaques from human infected skin (Sarfo et al., 2014). These early forms are characteristic of stages of *M. ulcerans* infection at which patients display painless symptoms without nerve damage. After 24 h, 98.3% of the injected dose is eliminated from the footpad. These quantities are consistent with the amounts detected in the tails of mice infected with *M. ulcerans* at the oedematous stage and with prior reports (Converse et al., 2014; Sarfo et al., 2014; Marion et al., 2016a,b). We can suggest three possible scenarios to explain this rapid decrease. (i) The diffusion of mycolactone in tissues away from the injection site. Our data suggest that this is highly unlikely, as we have shown that mycolactone was only detected in the inoculation site. (ii) The rapid degradation of mycolactone in tissues through enzymatic or chemical reactions. The validation of this hypothesis would require the detection of degradation or digestion products in tissues. As these products are currently unknown, the validation of this hypothesis will require in depth investigation. (iii) The diffusion of mycolactone from the tissue into the bloodstream. Our data suggest that this is possible, and are consistent with studies indicating mycolactone diffusion into peripheral blood in patients suffering from Buruli ulcers (Sarfo et al., 2011). However, the concentration of mycolactone in the bloodstream remained low, as no mycolactone was detected in the context of *M. ulcerans* infection in our mouse model and was insufficient to have a systemic immunosuppressive effect in the context of *M. ulcerans* infection.

We show here that mycolactone disappears rapidly from the tissue into which it is injected. No study to date, including this one, has reported a method for the efficient extraction of mycolactone from tissues (Sarfo et al., 2013; Converse et al., 2014). This represents a major obstacle to the development of a rapid and specific diagnostic tool based on mycolactone extraction from patient tissues.

Of importance, the residual dose of mycolactone was low but sufficient for hypoesthesia suggesting that mycolactone still ought to be considered as a drug candidate against pain. To this end, on one hand, mycolactone-like molecules, such as mycolactone 5b were investigated as drug candidates against chronic skin inflammation (Guenin-Macé et al., 2015, reviewed by Sarfo et al., 2016). On the other hand, encapsulation of mycolactone into polymers, which are able to improve bioavailability of poorly soluble drugs, would represent an exciting way to obtain stronger and more prolonged *in vivo* analgesic effects (Senanayake et al., 2014). In conclusion, owing to the use of optimization strategies, mycolactone would represent a promising analgesic that may be useful for the unmet needs in pain management.

## Ethics Statement

All animal experiments were performed in accordance with national (articles R214-87 to R214-90 from the French “rural code”) and European guidelines (directive 2010/63/EU of the European Parliament and of the Council of September 22, 2010 on the protection of animals used for scientific purposes). All protocols were approved by the Ethics Committee of the Pays de la Loire region under protocol CEEA 2015121410496026. Animals were maintained under specific pathogen-free conditions in the animal house of Angers University Hospital, Angers, France (agreement A 49 007 002).

## Author Contributions

JB, LM, EM, PR, DB, PB, and DG conceived and designed the experiments. JB, DB, J-PSA, AC, LM, and EM performed the experiments. JB, EM, M-LR, PR, and LM performed the data analysis. JB, M-LR, and LM wrote the manuscript.

## Conflict of Interest Statement

The authors declare that the research was conducted in the absence of any commercial or financial relationships that could be construed as a potential conflict of interest.
