# Supplementary material for: Mycolactone as Analgesic: Subcutaneous Bioavailability Parameters
Source: Front Pharmacol. 2019 Apr 12;10:378. doi: 10.3389/fphar.2019.00378 (PMC6473063; doi:10.3389/fphar.2019.00378)
Supplement: Supplementary file 1 [file Image_1.pdf]

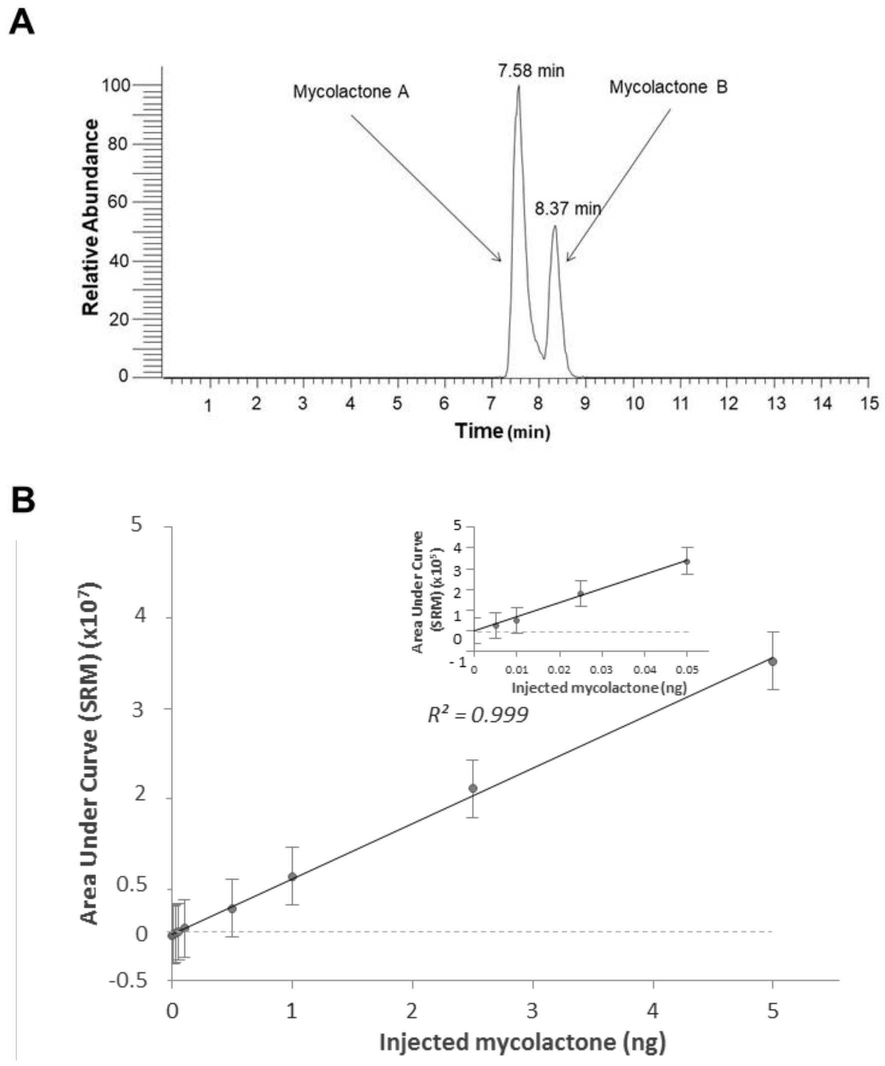

Supplementary figure 1: Detection and quantification of mycolactone by liquid chromatography coupled to tandem mass spectrometry

(A) Total ion current (TIC) for mycolactone A/B in selected reaction monitoring (SRM) (MS/MS: 765.5/429.2 UMA) (B) calibration curve.
